# Foliar Application of Nano, Chelated, and Conventional Iron Forms Enhanced Growth, Nutritional Status, Fruiting Aspects, and Fruit Quality of Washington Navel Orange Trees (*Citrus sinensis* L. *Osbeck*)

**DOI:** 10.3390/plants10122577

**Published:** 2021-11-25

**Authors:** Sherif F. El-Gioushy, Zheli Ding, Asmaa M. E. Bahloul, Mohamed S. Gawish, Hanan M. Abou El Ghit, Adel M. R. A. Abdelaziz, Heba S. El-Desouky, Rokayya Sami, Ebtihal Khojah, Taghred A. Hashim, Ahmed M. S. Kheir, Reda M. Y. Zewail

**Affiliations:** 1 Haikou Experimental Station, Chinese Academy of Tropical Agricultural Sciences (CATAS), Haikou 571101, China; sherif.elgioushy@fagr.bu.edu.eg; 2 Horticulture Department, Faculty of Agriculture (Moshtohor), Benha University, Toukh 13736, Egypt; 3 Department of Agricultural Economics, Faculty of Agriculture (Moshtohor), Banha University, Toukh 13736, Egypt; asmaa.bahlol@fagr.bu.edu.eg; 4 Pomology Department, Faculty of Agriculture, Damietta University, Damietta 34511, Egypt; msagawishaa@gmail.com; 5 Botany and Microbiology Department, Faculty of Science, Helwan University, Cairo 11111, Egypt; hanan8760@yahoo.com; 6 Central Lab of Organic Agriculture, Agricultural Research Center, Giza 12619, Egypt; dr.adel.organic@gmail.com; 7 Botany Department, Faculty of Agriculture (Moshtohor), Benha University, Toukh 13736, Egypt; Heba.alabd@fagr.bu.edu.eg (H.S.E.-D.); reda.zewail@fagr.bu.edu.eg (R.M.Y.Z.); 8 Department of Food Science and Nutrition, College of Sciences, Taif University, P.O. Box 11099, Taif 21944, Saudi Arabia; rokayya.d@tu.edu.sa (R.S.); eykhojah@tu.edu.sa (E.K.); 9 Soil and Water Department, Faculty of Agriculture (Moshtohor), Benha University, Toukh 13736, Egypt; taghreed.hashem@fagr.bu.edu.eg; 10 International Center of Biosaline Agriculture, ICBA, Dubai 14660, United Arab Emirates; 11 Soils, Water and Environment Research Institute, Agricultural Research Center, Giza 12112, Egypt

**Keywords:** nano-Fe, Fe-chelated (EDTA), TSS, fruiting aspects, fruit shape, navel orange, nutritional status, shelf life

## Abstract

Iron (Fe) is required for most metabolic processes, including DNA synthesis, respiration, photosynthesis, and chlorophyll biosynthesis; however, Fe deficiency is common in arid regions, necessitating additional research to determine the most efficient form of absorbance. Nano-fertilizers have characteristics that are not found in their traditional equivalents. This research was implemented on Washington navel orange trees (*Citrus sinensis* L. *Osbeck*) to investigate the effect of three iron forms—nano (Fe-NPs), sulfate (FeSO_4_), and chelated (Fe-chelated)—as a foliar spray on the growth, fruiting aspects, and nutritional status of these trees compared to control. The highest values of the tested parameters were reported when the highest Fe-NPs level and the highest Fe-chelated (EDTA) rate were used. Results obtained here showed that the spraying of the Washington navel orange trees grown under similar environmental conditions and horticulture practices adopted in the current experiment with Fe-NPs (nanoform) and/or Fe-chelated (EDTA) at 0.1% is a beneficial application for enhancing vegetative growth, flower set, tree nutritional status, and fruit production and quality. Application of Fe-NPs and Fe-chelated (EDTA, 0.1%) increased yield by 32.0% and 25% and total soluble solids (TSS) by 18.5% and 17.0%, respectively, compared with control. Spraying Washington navel orange trees with nano and chelated iron could be considered a significant way to improve vegetative growth, fruit production, quality, and nutritional status while also being environmentally preferred in the arid regions.

## 1. Introduction

Citrus fruit has long been regarded as one of the world’s most important fruit crops, both in terms of planting area and yield volume [1]. Citrus has been cultivated and valued in Egypt for over 4000 years. Oranges are the primary fruit of the citrus fruit, accounting for roughly 70% of citrus production [2]. The Mediterranean countries are the top producers of oranges in the international fresh market. Egypt has topped the list of countries that export oranges, with exports totaling 1.7 million tons in 2019, accounting for 38% of global orange exports in 2019 [3]. Citrus fruits are economically significant in Egypt, with their large-scale production, estimated at 4.5 million tons per year, playing an important role in the region’s fruit economy. Citrus fruits have a variety of positive health and nutrient properties [4], due to their richly in vitamin C and folic acid, and they are free of sugar, sodium, and cholesterol. Citrus fruits’ potassium, calcium, folate, thiamine, niacin, vitamin B6, phosphate, magnesium, and copper levels may lower the risk of heart disease, various types of cancer, and respiratory system diseases, reducing the risk impacts of coronavirus pandemics like COVID-19 on humans [5,6,7].

Iron is required as a cofactor for several enzymes included in mitochondrial respiration, nucleic acid synthesis, photosynthesis, metal homeostasis, and protein, as well as functional and structural integrity and chlorophyll content [8,9,10]. Plants obtain Fe from the soil, and its availability in the form of Fe2^+^ is essential for their proper development and growth. Several physiological activities in the rhizosphere are hampered by the iron storage and availability in soil [11,12]. Fe deficiency (FDS) and Fe toxicity affect roughly 30% and 18% of the world’s soil, respectively [13,14,15]. Fe deficiency (FD) is common in alkaline soils; therefore, this phenomenon is prevalent in Egyptian soils, which are characterized by higher alkalinity, trace element pollution, nitrate, climate variability, and poor aeration. The majority of Fe in the soil is unavailable and cannot be used by plant roots [16]. Furthermore, reduced mobility of the Fe-carrying system restricts chlorophyll production due to a decline in the mitochondria cell charges for the excretion of mugineic acid (MA) [17,18]. FDS is a significant concern all over the world, with the bulk of cases occurring in the United States, East and West Africa, and certain parts of Europe and Asia [19,20]. Consequently, applying the appropriate dose and form of Fe via foliar application need further research, to reduce plant deficiency; the use of Fe in traditional mixed fertilizers remains the most common method of increasing crop yields; however, it is frequently ineffective due to poor nutrient-use performance [21]. Chelated Fe (Che-Fe) was proposed as an alternative method to improve Fe absorption by growing plants [10,22]. However, the use of chelated Fe in various doses on citrus has received less attention thus far, particularly in arid regions. The word nanofertilizer refers to a nanomaterial that is either a plant nutrient (micro-or macronutrients) or a transporter of a plant nutrient [23]. Due to their small size and large surface area, such nanomaterials have unique optical, physical, and biological properties [24]. The purpose of developing nanoparticle fertilizers is to effectively deliver required nutrients to plants while not adding large amounts of fertilizer to the environment [25]. In agriculture, nanotechnology has been studied to reduce the use of reactive chemicals, reduce nutrient losses, and increase economic crop yields through precise nutrient management [26], followed by micronutrients and amino acids [27]. Other studies found that application of nanoparticles (NPs) enhanced iron content, redox, growth, and yield of *B*. *juncea* [28]; improved growth, yield, and quality of rice [29]; improved the biological activities of sweet basil [30]. Nonetheless, the impact of foliar nanoiron (Fe-NPs) as a replacement for conventional Fe on citrus yield and quality in arid regions is unknown. Therefore, the main objective of this research is to explore the effects of different iron forms—chelated, conventional, and nanoforms—on the growth performance, fruit yield, quality, and shelf life of navel orange trees (*Citrus sinensis*) in arid regions.

## 2. Results

### 2.1. Vegetative Growth Measurements

Table 1 shows the influence of iron (nano, sulfate, and chelated) foliar spray on some vegetative growth parameters of Washington navel orange trees (*Citrus sinensis* L. Osbeck) during 2019 and 2020. The investigated parameters were the number of developed shoots per meter of each tagged main branch (limb/scaffold), average shoot length and diameter, number of leaves per shoot, average leaf area, and assimilation area per shoot. The responses of the aforementioned parameters to the various tested iron forms showed significant variation. The highest values were significantly associated with Washington navel orange trees that were subjected to foliar Fe-NPs2 spraying (T3: 1/40 of the Fe-NPs stock), followed by 0.1% Fe-chelated (T7) and Fe-NPs1 (T2: 1/80 of the Fe-NPs stock) treatments, respectively, and generally, they produced similar results during both experimental seasons. During both 2019 and 2020 experimental seasons, the lowest values were typically observed for the water-sprayed Washington navel orange trees (control), with statistical significance. Furthermore, the results of three additional nutritive compounds tested fell between the two extremes mentioned above. Despite statistical significance when compared with the above-mentioned superior and inferior treatments during the two experimental seasons, these three nutritive compound treatments did not differ significantly from one another. Overall, it was possible to conclude that most treatments exhibited significant increases in the six studied growth parameters when compared with the control (water spraying). T3 was statistically the most effective treatment, followed by the seventh and second treatments, whereas the iron forms of FeSO_4_ (T4 and T5) produced inferior results. The results of the remaining treatments were in the middle of these ranges, with varying degrees of responsiveness among growth measurements.

### 2.2. Chlorophyll, Carotenoid, Macro and Micronutrient Content in Orange Tree Leaves

The data in Table 2, Table 3 and Table 4 show that the different investigated treatments affected the levels of chlorophyll A and B, carotenoids, and total chlorophyll, as well as the contents of N, P, K, Ca, Mg, Fe, Mn, and Zn (nutritional status) in the leaves of Washington navel orange trees during the 2019 and 2020 experimental seasons. In terms of the effect of foliar spraying with the various investigated iron forms (nano, sulfate, and chelated), data obtained during both seasons revealed that all treatments significantly increased all investigated leaf chemical compositions when compared with the control. With the exception of the application of FeSO_4_ at a lower level (0.1 percent), such a trend was observed during both seasons, whereas a slight increase was observed for most chemical parameters, with a few exceptions related to the leaf P. (during the two seasons). Furthermore, foliar spraying with Fe-NPs_2_ (T3: 1/40 of the Fe-NPs stock) produced the highest concentrations of chlorophylls A and B, carotenoids, total chlorophyll, and macro-and micronutrients in the leaves. Furthermore, as a foliar spray, 0.1 percent Fe-chelated (T7) ranked second statistically, followed by Fe-NPs1 (T2). This trend was observed throughout the two seasons, with the exception of P percent in leaves, which was the opposite. In contrast, the lowest levels of all or most leaf chemical constituents were typically found in the control (T1) and spraying treatments with both FeSO_4_ concentrations (0.1% and 0.2%). Finally, the outcomes for other treatments fell somewhere between the two extremes mentioned above.

### 2.3. Fruit Aspect and Shelf Life

All of the fruiting measurements (i.e., fruiting aspects, physical, and chemical properties) clearly responded to all of the investigated treatments (Table 5, Table 6 and Table 7), and the degree of response varied from one fruiting measurement to another. Furthermore, the rate of difference in such fruiting measurements produced by Fe-NPs was greater than that produced by the FeSO_4_ and Fe-chelated forms. However, when compared with the control (water spray) and other Fe forms, the two Fe-NPs spray solutions significantly increased all fruiting measurements. With only two exceptions, the response of these fruiting measurements to Fe-NPs revealed clearly that the highest values of such measurements were significantly correlated, with the higher Fe-NPs_2_ spray solution (1/40 dilution of the Fe-NPs solution). Furthermore, the Fe-NPs1 (T2) spray solution was statistically superior to the foliar spray treatment with 0.1 percent Fe-chelated (T7). The smallest increase over the control, on the other hand, was always associated with a lower FeSO_4_ level (0.1%). The differences between the various investigated treatments were significant during both seasons, with few exceptions; in particular, their effect did not reach the level of significance. Regarding the exceptions found for the response of both fruit shape index (polar equatorial diameter) and fruit juice total acidity, they may be logically explained by two facts—the fruit shape index may be attributed to a similar rate of the response of both fruit dimensions to a given spray treatment, whereas the increase in fruit juice total acidity was interpreted as a dilution effect caused by the increase in fruit juice content or a sign of earlier maturation by all spraying treatments.

**Table 1:** Influence of iron (nano, sulfate, and chelated) foliar spray on some vegetative growth parameters of Washington navel orange trees during 2019 and 2020 experimental seasons.

Parameters Treatments	No. of New Shoots (NS)		Shoot Length, cm (SL)		Shoot Diameter, mm (SD)		No. of Leaves/Shoot (NOL)		Leaf Area, cm^2^ (LA)		Assimilation Area, m^2^/Shoot (TAA)	
	2019	2020	2019	2020	2019	2020	2019	2020	2019	2020	2019	2020
T1	23.33 f	23.33 d	35.67 d	36.33 e	3.12 b	3.14 e	39.67 c	40.67 c	17.55 c	17.59 c	6.96 c	7.15 c
T2	27.00 cd	27.67 b	41.33 b	41.33 bc	3.23 ab	3.22 c	43.33 b	44.00 b	17.70 ab	17.71 a	7.67 b	7.79 b
T3	33.00 a	32.67 a	45.67 a	44.33 a	3.33 a	3.29 a	46.67 a	46.33 a	17.73 a	17.71 a	8.27 a	8.20 a
T4	24.00 ef	23.33 d	37.00 cd	37.33 e	3.22 ab	3.14 e	39.67 c	41.00 c	17.58 c	17.59 bc	6.97 c	7.21 c
T5	25.67 de	25.33 c	39.33 bcd	39.00 d	3.18 ab	3.18 d	43.33 b	43.33 b	17.66 b	17.67 ab	7.65 b	7.66 b
T6	27.67 bc	27.00 bc	40.33 bc	40.33 cd	3.23 ab	3.21 c	44.33 b	43.67 b	17.71 a	17.71 a	7.85 b	7.73 b
T7	29.33 b	28.67 b	43.33 ab	42.33 b	3.28 ab	3.24 b	46.33 a	46.00 a	17.71 a	17.73 a	8.21 a	8.15 a

Means followed by the same letter/s within each column did not significantly differ at 5% level. T1, T2, T3, T4, T5, T6, and T7 are control, Fe-NPs 1, Fe-NPs 2, FeSO_4_ (0.1%), FeSO_4_ (0.2%), Fe-chelated (0.05%), and Fe-chelated (0.1%), respectively.

**Table 2:** Influence of iron (nano, sulfate, and chelated) foliar spray on chlorophyll (A), chlorophyll (B), carotenoids, and total chlorophyll of Washington navel orange trees during 2019 and 2020 experimental seasons.

Parameters Treatments	Chlorophyll A, mg/g-1F.W. (CA)		Chlorophyll B, mg/g-1F.W. (CB)		Total Chlorophyll, mg/g-1F.W. (TC)		Carotenoids, mg/g-1F.W. (CAR)	
	2019	2020	2019	2020	2019	2020	2019	2020
T1	6.45 c	6.52 d	3.18 d	3.00 d	9.63 d	10.59 e	3.04 d	3.11 e
T2	7.56 ab	7.31 bc	4.16 b	4.05 b	11.72 b	11.12 b	3.78 b	3.96 b
T3	8.04 a	8.08 a	4.53 a	4.60 a	12.57 a	11.62 a	4.04 a	4.15 a
T4	7.15 b	7.15 c	3.70 c	3.65 c	10.8 c	10.79 d	3.16 d	3.26 d
T5	7.94 a	7.62 b	3.81 c	3.95 b	11.75 b	10.96 c	3.48 c	3.60 c
T6	7.96 a	7.96 a	4.11 b	4.05 b	12.07 ab	10.99 c	3.89 ab	3.96 b
T7	8.00 a	8.01 a	4.24 ab	4.18 b	12.24 ab	11.02 c	3.98 a	4.03 ab

Means followed by the same letter/s within each column did not significantly differ at 5% level. T1, T2, T3, T4, T5, T6, and T7 are control, Fe-NPs 1, Fe-NPs 2, FeSO_4_ (0.1%), FeSO_4_ (0.2%), Fe-chelated (0.05%), and Fe-chelated (0.1%), respectively.

**Table 3:** Influence of iron (nano, sulfate, and chelated) foliar spray on N, P, K, Mg, and Ca percentages of Washington navel orange trees during 2019 and 2020 experimental seasons.

Parameters Treatments	N (%)		P (%)		K (%)		Mg (%)		Ca (%)	
	2019	2020	2019	2020	2019	2020	2019	2020	2019	2020
T1	2.73 e	2.76 f	0.163 a	0.163 a	1.66 d	1.64 e	0.565 c	0.544 e	4.50 e	4.53 d
T2	2.95 c	3.04 b	0.162 ab	0.158 b	1.84 b	1.87 b	0.6095 b	0.592 c	4.73 b	4.73 b
T3	3.04 a	3.14 a	0.160 abc	0.158 b	1.96 a	1.99 a	0.6355 ab	0.633 a	4.87 a	4.83 a
T4	2.90 d	2.83 e	0.158 bcd	0.151 c	1.71 d	1.73 d	0.579 c	0.572 d	4.57 de	4.56 d
T5	2.94 cd	2.92 d	0.157 cde	0.155 bc	1.78 c	1.79 cd	0.615 ab	0.597 c	4.64 c	4.65 c
T6	2.98 bc	2.99 c	0.155 de	0.158 b	1.78 c	1.83 bc	0.624 ab	0.613 b	4.64 cd	4.74 b
T7	3.01 ab	3.04 b	0.154 e	0.163 a	1.86 b	1.87 b	0.638 a	0.627 a	4.73 b	4.75 b

Means followed by the same letter/s within each column did not significantly differ at 5% level. T1, T2, T3, T4, T5, T6, and T7 are control, Fe-NPs 1, Fe-NPs 2, FeSO_4_ (0.1%), FeSO_4_ (0.2%), Fe-chelated (0.05%), and Fe-chelated (0.1%), respectively.

**Table 4:** Influence of iron (nano, sulfate, and chelated) foliar spray on Fe, Mn, and Zn (ppm) contents of Washington navel orange trees during 2019 and 2020 experimental seasons.

Parameters Treatments	Fe (ppm)		Mn (ppm)		Zn (ppm)	
	2019	2020	2019	2020	2019	2020
T1	76.08 f	75.17 e	44.17 d	43.94 d	29.03 b	27.60 g
T2	83.10 b	83.03 b	46.98 b	46.42 b	35.08 a	31.62 b
T3	85.64 a	85.11 a	48.37 a	47.51 a	35.97 a	32.38 a
T4	77.86 e	76.37 de	44.86 cd	43.90 d	32.23 ab	28.36 f
T5	79.23 d	77.27 d	45.14 c	44.48 cd	30.25 ab	29.14 e
T6	81.27 c	80.49 c	45.59 c	45.31 c	31.94 ab	29.87 d
T7	82.52 b	81.58 c	47.31 b	46.31 b	33.52 ab	30.28 c

Means followed by the same letter/s within each column did not significantly differ at 5% level. T1, T2, T3, T4, T5, T6, and T7 are control, Fe-NPs 1, Fe-NPs 2, FeSO_4_ (0.1%), FeSO_4_ (0.2%), Fe-chelated (0.05%), and Fe-chelated (0.1%), respectively.

**Table 5:** Influence of iron (nano, sulfate, and chelated) foliar spray on some fruiting aspects of Washington navel orange trees during 2019 and 2020 experimental seasons.

Parameters Treatments	Fruit Set, % (FS)		Fruit Retention, % (FR)		Average Fruit Weight, g (AFW)		No. of Fruits/Tree (NOFT)		Yield (kg)/Tree (FWT)		Yield, t ha-1 (YPF)	
	2019	2020	2019	2020	2019	2020	2019	2020	2019	2020	2019	2020
T1	17.91 e	17.22 d	12.44 e	12.07 d	261.0 b	265.33 d	155.00 f	160.33 f	40.45 f	42.55 e	25.16 e	26.87 d
T2	19.68 b	18.95 ab	14.56 b	13.91 b	269.67 ab	272.00 bc	175.67 b	178.00 b	47.38 bc	48.42 b	30.42 bc	31.37 b
T3	20.108 a	19.287 a	15.97 a	14.67 a	276.67 a	281.00 a	186.67 a	184.33 a	51.65 a	51.82 a	34.01 a	34.68 a
T4	18.19 e	17.15 d	13.38 d	12.39 d	268.33 ab	269.33 cd	162.33 e	165.33 e	43.52 e	44.53 d	27.79 d	28.56 cd
T5	18.64 d	18.357 c	13.98 c	12.83 c	266.33 ab	273.67 bc	171.67 cd	169.33 d	45.73 d	46.34 c	28.99 cd	30.18 bc
T6	18.74 d	18.59 bc	14.87 b	13.67 b	273.33 ab	271.33 bcd	169.33 d	173.33 c	46.29 cd	47.03 bc	30.11 bc	30.37 b
T7	19.17 c	18.98 a	15.62 a	13.97 b	276.33 a	276.00 ab	175.33 bc	183.67 a	48.46 b	50.70 a	31.87 ab	33.32 a

Means followed by the same letter/s within each column did not significantly differ at 5% level. T1, T2, T3, T4, T5, T6, and T7 are control, Fe-NPs 1, Fe-NPs 2, FeSO_4_ (0.1%), FeSO_4_ (0.2%), Fe-chelated (0.05%), and Fe-chelated (0.1%), respectively.

**Table 6:** Influence of iron (nano, sulfate, and chelated) foliar spray on some fruit physical properties of Washington navel orange trees during 2019 and 2020 experimental seasons.

Parameters Treatments	Peel Diameter, mm (PT)		Polar Diameter, cm (PD)		Equatorial Diameter, cm (ED)		Fruits Shape Index (FSI)		Juice Weight, g (JW)		Juice, % (J)	
	2019	2020	2019	2020	2019	2020	2019	2020	2019	2020	2019	2020
T1	3.103 c	3.113 d	8.310 a	8.293 d	8.323 b	8.327 e	0.998 a	0.996 b	109.26 c	109.57 e	41.86 c	41.29 d
T2	3.127 b	3.130 c	8.347 a	8.327 b	8.373 a	8.350 bcd	0.997 a	0.997 ab	113.87 abc	113.98 bc	42.22 bc	41.90 bc
T3	3.167 a	3.170 a	8.350 a	8.353 a	8.367 ab	8.367 ab	0.998 a	0.998 a	118.92 a	119.49 a	42.98 a	42.52 a
T4	3.107 c	3.113 d	8.330 a	8.310 c	8.360 ab	8.333 de	0.996 a	0.997 ab	112.32 bc	111.08 de	41.86 c	41.24 d
T5	3.133 b	3.130	8.347 a	8.320 bc	8.373 a	8.340 cde	0.997 a	0.998 ab	111.98 bc	114.13 bc	42.04 bc	41.70 c
T6	3.160 a	3.113 d	8.340 a	8.357 a	8.370 a	8.380 a	0.996 a	0.997 ab	115.81 ab	113.50 cd	42.37 b	41.83 c
T7	3.170 a	3.157 b	8.347 a	8.333 b	8.370 a	8.357 bc	0.997 a	0.997 ab	116.97 ab	116.23 b	42.33 b	42.11 b

Means followed by the same letter/s within each column did not significantly differ at 5% level. T1, T2, T3, T4, T5, T6, and T7 are control, Fe-NPs 1, Fe-NPs 2, FeSO_4_ (0.1%), FeSO_4_ (0.2%), Fe-chelated (0.05%), and Fe-chelated (0.1%), respectively.

**Table 7:** Influence of iron (nano, sulfate, and chelated) foliar spray on some fruit chemical properties and shelf life of Washington navel orange trees during 2019 and 2020 experimental seasons.

Parameters Treatments	T.S.S., % (TSS)		Total Acidity, % (TA)		TSS/Acid Ratio (TS/AC)		Total Sugars, % (TS)		V.C. (VC)		Shelf Life, Days (SLIF)	
	2019	2020	2019	2020	2019	2020	2019	2020	2019	2020	2019	2020
T1	11.13 c	11.60 d	1.044 a	1.062 a	10.66 c	10.92 e	8.68 b	9.59 c	57.31 e	59.12 e	13.60 d	16.33 e
T2	12.87 ab	13.17 b	0.988 b	0.988 b	13.03 b	13.32 c	9.07 ab	10.11 bc	62.66 bc	63.36 b	23.00 ab	23.67 bc
T3	12.89 ab	13.91 a	0.907 d	0.903 d	14.22 a	15.40 a	9.62 a	10.95 a	63.79 a	64.92 a	25.33 a	26.33 a
T4	12.34 b	11.82 d	0.9737 b	1.000 b	12.68 b	11.82 d	9.34 ab	9.73 c	60.64 d	60.29 d	15.67 cd	17.33
T5	12.59 ab	12.27 c	0.930 cd	0.950 c	13.54 ab	12.92 c	9.43 ab	10.04 bc	62.09 c	61.00 d	18.00 c	20.33 d
T6	12.41 ab	12.57 c	0.938 c	0.954 c	13.23 b	13.17 c	9.34 ab	10.07 bc	62.19 c	62.12 c	20.67 b	22.33 cd
T7	13.22 a	13.24 b	0.918 cd	0.934 c	14.40 a	14.17 b	9.70 a	10.48 ab	62.98 b	63.36 b	22.67 b	25.33 ab

Means followed by the same letter/s within each column did not significantly differ at 5% level. T1, T2, T3, T4, T5, T6, and T7 are control, Fe-NPs 1, Fe-NPs 2, FeSO_4_ (0.1%), FeSO_4_ (0.2%), Fe-chelated (0.05%), and Fe-chelated (0.1%), respectively.

**Figure 1:**
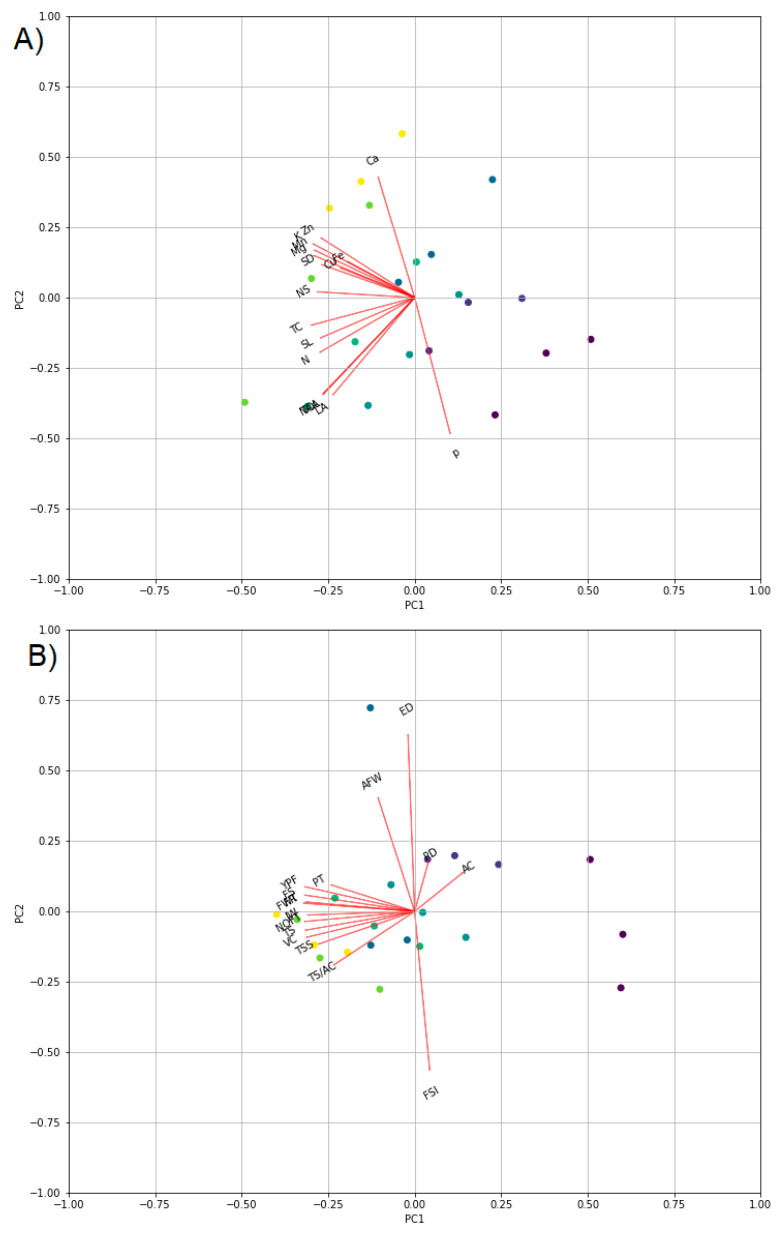
Principal component analysis (PCA) to show the correlation between treatments (scores) and crop features (loadings). A represents PCA for the corresponding treatments and the vegetative growth, chlorophyll, and mineral contents, while B represents PCA for the corresponding treatments and fruit aspects and quality. Treatments are control, Fe-NPs 1, Fe-NPs 2, FeSO_4_ (0.1%), FeSO_4_ (0.2%), Fe-chelated (0.05%), and Fe-chelated (0.1%) for T1, T2, T3, T4, T5, T6, and T7, respectively. The treatments were gradually colored from dark blue (control) to green (T7). The vegetative growth features and minerals (**A**) included No. of new shoots (NS), shoot length (SL), shoot diameter (SD), No. of leaves per shoot (NOL), leaf area (LA), assimilation area (TAA), total chlorophyll (TC), chlorophyll A (CA), chlorophyll B (CB), carotenoids (CAR), nitrogen (N), phosphorus (P), potassium (K), calcium (Ca), magnesium (Mg), iron (Fe), manganese (Mn), and zinc (Zn). The yield and quality aspects (**B**) included fruit set (FS), fruit retention (FR), No. of fruits per tree (NOFT), average fruit weight (AFW), yield per tree (FWT), total yield (YPF), peel diameter (PT), polar diameter (PD), equatorial diameter (ED), fruit shape index (FSI), juice weight (JW), juice, % (J), total soluble solids (TSS), Total acidity, % (TA), TSS/acid ratio (TS/AC), total sugars (TS), (V.C.) and shelf life (SLIF).

**Figure 2:**
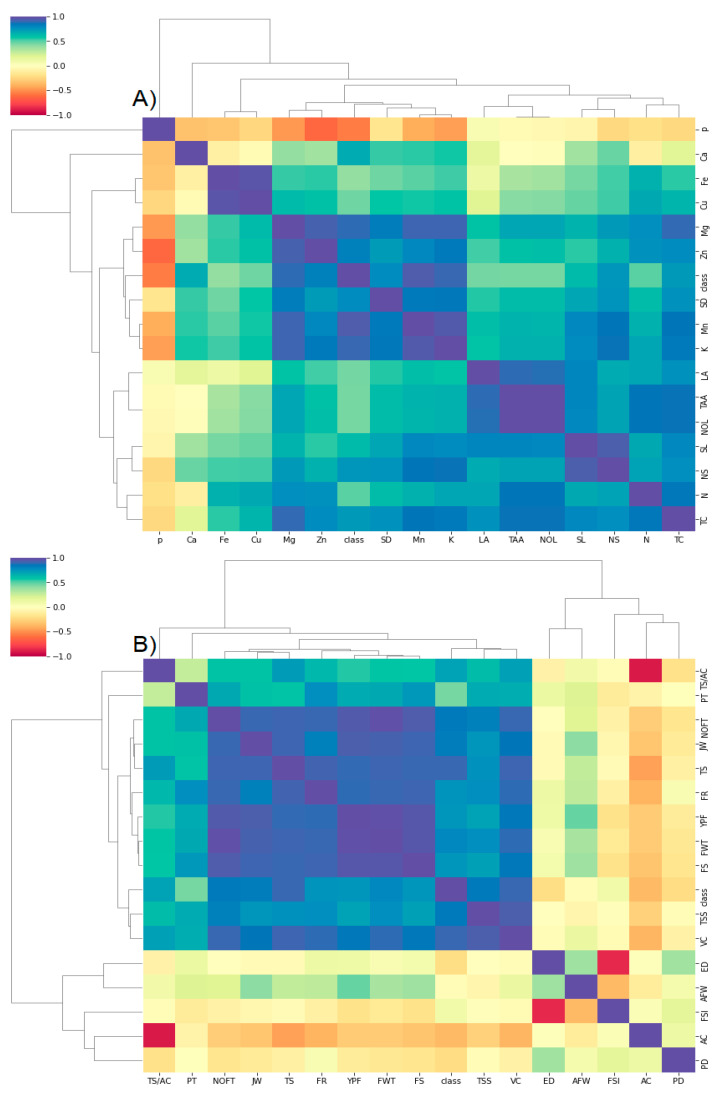
Heatmap correlation matrix for the vegetative growth, chlorophyll, and mineral contents (**A**), as well as the fruit aspects and quality (**B**).

The results of (SEM) revealed that the iron nanoparticles had a needle-like shape, as shown in Supplementary Figure S1, which shows the Fe-NP diameter and length. The particle diameter was in the 21 ± 9 nm range, and the length was 87 ± 1 nm. The morphology and size of the particles were also determined using a transmission electron microscope (TEM, JEOL GEM-1010 transmission electron microscope at 70 kV) and are included in the supplementary data. The zeta potential of iron nanoparticles was −12.1 mV, indicating that the nanoparticles were stable (Supplementary Figure S3). The average size of Fe-NPs was 20.83 nm, with 9.48 percent of all particles distribution, which was consistent with the result obtained using SEM, which was 209. Principle component analysis (PCA) and correlation heatmap were used to better understand the relationships between the corresponding treatments and yield and quality of Washington navel orange trees (Figure 1 and Figure 2).

## 3. Discussion

Because iron is required for metabolic processes such as DNA synthesis, respiration, and photosynthesis, it is an essential micronutrient for all living creatures [31,32]. Furthermore, because iron is a prosthetic group constituent of many enzymes such as cytochromes in the electron transport chain, it is required for many biological tasks [33]. It also participates in chlorophyll synthesis, so it is required for the chloroplast’s structure and function. Iron deficiency is a common phenomenon in arid regions and most of the soluble Fe in the soil is complexed by natural organic compounds due to higher content of calcium carbonate and pH, variability in temperature, and poor aeration [34,35]. Nanofertilizers could be utilized to improve traditional agricultural techniques and provide sustainable development by reducing agricultural input waste and improving management and conservation strategies [36,37]. The highest values of vegetative growth features were significantly associated with Washington navel orange trees (*Citrus sinensis*) that were subjected to the third treatment in this study (Fe-NPs_2_). This may be attributed to the advantages of nanoparticles in agrochemical delivery due to their large surface area, ease of attachment, and rapid mass transfer [38]. Furthermore, during the two experimental seasons, both the chelated iron (0.1%) and the second treatment (Fe-NPs1) produced the same significant effect. Our findings were similar to those obtained by [39] for vine (*Vitis vinifera*). They revealed that Fe_2_O_3_ NPs at 1% and some forms such as nanocalcite (CaCO_3_ 40%), nano-SiO2 (4%), andMgO (1%) improved Ca, Mg, and Fe uptake and significantly improved P intake with micronutrients Zn and Mn. Furthermore, Kah [40] reported that the efficacy of nano-agrochemicals is up to 30% higher than the conventional products. Susceptibility to Fe chlorosis is determined by a plant’s response to Fe deficiency stress, which is genetically controlled [41]. Existing Fe chlorosis can be corrected with organic chelates because they provide sufficient Fe at lower rates than inorganic Fe sources. This could explain why we integrated different forms of nano and chelated iron, to maximize the efficiency and to explore the optimum form, i.e., due to its efficiency, compared with other application forms [41], foliar application of Fe forms was used and recommended to correct Fe chlorosis in citrus trees. The study by [42] revealed that because Fe NPs are naturally nontoxic, they have been used as Fe-enriching fertilizers to replenish Fe content in plants, proving the importance of using this material in the current study. As regards the effect of the different investigated iron spray solutions (nano, sulfate, and chelated), the data obtained during both seasons revealed that all treatments significantly increased all investigated leaf chemical composition parameters, compared with the control. This could be attributed to Fe interfering with the structural and catalytic components of proteins and enzymes, which are required for the normal development of pigment biosynthesis and photosynthesis activation [43]. Furthermore, Fe is essential for enhancing photosynthesis processes and improving carbohydrate translocation to different parts of the radish plant (*Raphanus sativus*) [44]. Translocation enhancement could indirectly increase the biosynthesis of total phenols, flavonoids, and tannins in plants treated with ZnO and FeO GNP fertilizers. Several studies have found that Fe, in its natural or nanoform, improves leaf photosynthetic pigments and photosynthesis parameters [45,46,47].

Due to the preliminary improvement of vegetative growth and photosynthesis in response to the application of different iron forms (nano, sulfate, and chelated), the fruit aspects and shelf life improved significantly. A few studies [48,49] provided scientific evidence for increased fruit quality and yield in many crops using Fe fertilization. Furthermore, iron nanoparticles improved plant stability under drought stress, increased the quantity and quality of plantlets’ morphological and growth-related characteristics in vitro, and compensated for the negative effects of drought stress in vitro strawberry (*Fragaria ananassa*) cultivation [50]. Nonetheless, the application of various forms of Fe on citrus in arid regions has received less attention thus far, demonstrating the importance of the current study.

Principal component analysis (PCA) was used to better understand the relationship between different treatments and crop features such as vegetative, growth, mineral, and fruit aspects (Figure 1). There was an entirely positive correlation between contents of Ca, Fe, Cu, Zn, K, Mn, Mg, NS and SD, which were also correlated positively with treatments T3, T7, and T6. Meanwhile, there were negative correlations between such parameters and LA, NOL, SL, TAA, TC, CA, CB, CAR, and content of N, and P (Figure 2A). The features TC, SL, N, LA, NOL, CA, CB, CAR, and N had positive correlations with treatments T4 and T5, but the content of P had a positive correlation with control (T1) and negative correlations with other treatments (Figure 2A). Regarding the fruit yield and quality, the features FS, FR, NOFT, FWT, YPF, PT, JW, TSS, TS, and SLIF had positive correlations between each other and with treatments T3, T7, T6, T5, and T4. Meanwhile, there was a negative correlation between such features and AC, PD, ED, AFW, and FSI, which correlated positively with treatments T1 and T2 (Figure 2B). Finally, the treatments Fe-NPs2 (T3) and Fe-chelated, 0.1% (T7) outperformed the others in terms of most vegetative, growth, yield, and quality of Washington navel orange trees grown in arid and semi-arid conditions. These findings were also supported by the correlation matrix (Figure 2).

The correlation matrix interpreted the relationships between vegetative growth, chlorophyll, and minerals, as well as yield and quality (Figure 2). Most mineral contents, with the exception of Ca, P, and chlorophyll characteristics, showed strong positive correlations with each other (Figure 2A). Furthermore, yield and quality characteristics such as FS, FR, NOFT, FWT, YPF, PT, JW, TSS, TS, and SLIF correlated positively with each other and negatively with other characteristics such as AC, PD, ED, AFW, and FSI (Figure 2B). In conclusion, improving orange yield and quality does not necessitate ensuring the best aspects, confirming the importance of yield and quality over other factors.

## 4. Materials and Methods

### 4.1. Study Location, Climate Data, and Soil Properties

**Figure 3:**
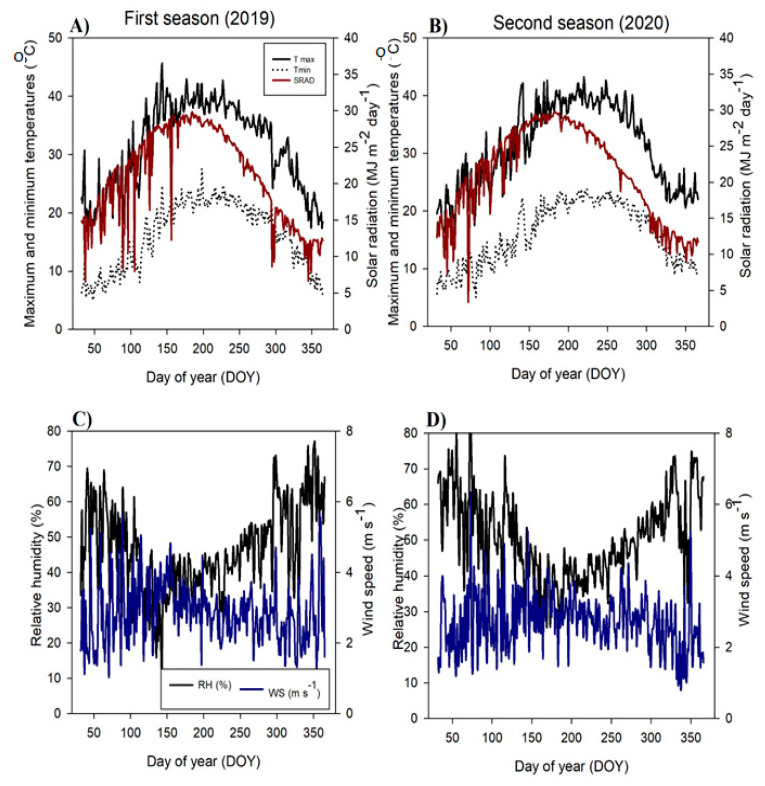
Daily climatic data as maximum temperature, minimum temperature, and solar radiation (**A**,**B**), as well as relative humidity and wind speed (**C**,**D**) over two seasons in the studied area.

This study was conducted on 11-year-old Washington navel orange trees budded on Sour orange rootstock planted 5 × 5 m apart under surface irrigation in a private orchard Qalubia Governorate, Egypt (30.4° N and 31.1° E), during the 2019 and 2020 seasons. All trees were received the same agronomic practices such as irrigation, fertilization, as well as weed and pest control, as recommended by the Ministry of Agriculture of Egypt. This study looked into the effects of foliar iron spraying (nano, sulfate, and chelated forms) on the growth, fruiting, and quality of the trees. A mechanical and chemical analysis of soil surface (0–60 cm) was implemented before the first season (2019) [51] (Supplementary Table S1). Daily climatic data included maximum and minimum temperature, solar radiation, relative humidity, and wind speed over two seasons, as presented in Figure 3.

### 4.2. Preparation of Green Fe-NPs

#### 4.2.1. Guava Leaf Extract

Fresh guava leaves (*Psidium guajava* L.) were collected from the Al-Qanater Horticultural Research Station, Qalubia Governorate, cleaned and washed with tap water first, then with distilled water, to remove the associated pollutants. The samples were air-dried for two weeks before being ground to a fine powder in the laboratory and used to make the extract. About 150 g of the powder sample was boiled in 1 L of distilled water for 20 min and filtered after cooling. The extracts were then kept at 4 °C until they were used to make green Fe-NPs.

#### 4.2.2. Green Synthesis of Fe-NPs

A solution of 5 mM FeSO_4_ was set through FeSO_4_ dissolving in the distilled water. Green synthesis using the *Psidium guajava* L. (guava) leaf extract and Fe ions were reduced and capped using the method was described by [52], with some modifications. These modifications included adding 200 mL of the extract to the aqueous solution of FeSO_4_ at normal atmospheric pressure, and the pH was adjusted to 9.0. To obtain the stock solution, the mixture was constantly stirred at 70 °C–80 °C for 8 h, followed by further stirring at room temperature overnight without heating. The prepared Fe-NPs solution was diluted 80 and 40 times to obtain Fe-NPS_1_ and Fe-NPS_2_, respectively, for application as a foliar spray with two different concentrations.

#### 4.2.3. Scanning Electron Microscopy (SEM)

Scanning electron microscopy was used to determine the morphology and size of the particles (Supplementary Figure S1). The shape and size of the iron nanoparticles were revealed by scanning electron microscopy measurements. The results showed that the iron nanoparticles had almost a needle shape, as clarified in Supplementary Figure S1, which displays the Fe NP diameter and length. The particle diameter was in the range of 21 ± 9 nm, and the length was 87 ± 1 nm.

#### 4.2.4. Transition Electron Microscope (TEM)

The morphology and size of the particles were determined by a Transmission electron microscope (TEM, JEOL GEM-1010 transmission electron microscope at 70 kV) at the Regional Center for Mycology and Biotechnology, Egypt. A drop containing Fe-NPs was deposited onto carbon-coated copper grids (CCGs) and then exposed to the infra light for 30 min. The micrograph was analyzed by JEOL—JEM 1010—Transmission Electron Microscope at 70 kV in the RCMB, Al-Azhar (Supplementary Figure S2).

#### 4.2.5. Zeta Potential and Dynamic Light Scattering

The zeta potential (surface charge) is important in determining the stability and shelf life of nanoparticles. A high zeta potential value, either positive or negative, is required to prevent particle aggregation [53]. A high zeta potential increased repulsive forces relative to attractive forces, preventing particle agglomeration [54]. The zeta potential value of iron nanoparticles was −12.1 mV, indicating good stability of the nanoparticles (Supplementary Figure S3). The size of Fe-NPs was 20.83 nm on average, with 9.48% of all particles distribution, which was in good agreement with the result obtained using SEM, which was 21 ± 9 (Supplementary Figure S4).

### 4.3. Treatments and Experimental Layout

This investigation was included seven applications for nano, chelated, and (FeSO_4)_ iron forms, and they are displayed in Table 8.

The seven investigated fertilization treatments were organized using a complete randomized block design with three replications, with a single tree representing one replicate. Consequently, 21 healthy fruitful trees were carefully chosen as being healthy and disease-free trees. The trees were divided into three categories (blocks) based on their growth vigor, with each block containing seven similar trees to receive the seven foliar spray treatments. Starting on 1 March of each season, the trees assigned to each treatment were sprayed with the specific solution five times at one-month intervals.

### 4.4. Measurements and Data Collection

In late March 2019 and 2020, four main branches (limbs/scaffolds) that were well distributed around the periphery of each tree were carefully selected and tagged during the 2019 and 2020 seasons, respectively. In addition, 20 new spring-developed shoots were also labeled.

#### 4.4.1. Vegetative Growth Measurements

In mid-October, the following vegetative growth parameters were assessed during the 1st and 2nd experimental seasons, respectively. The average number of newly developed shoots per 1 m of every tagged limb, the average length and diameter, the number of leaves per labeled shoot, and the average leaf area (cm^2^) on a weight basis were estimated. Hence, 20 mature leaves from the previously labeled shoots per limb were randomly collected. Subsequently, 20 disks of 1 cm each were collected and oven-dried, together with the remaining leaves at 80 °C, until a constant weight was reached. The average leaf area in cm^2^ was determined using the dry weight of a specific surface area of leaves, such as 20 leaf discs and the total weight of 20 leaves. In addition, the assimilation area per shoot was determined by multiplying leaf area by the number of leaves per shoot.

#### 4.4.2. Nutritional Status Measurements and Leaf Mineral Composition

The leaf chlorophylls A and B and carotenoid content in response to the different tested treatments during both seasons were determined as described by [55]. In total, 20 fresh mature leaves were collected from the middle portion of the labeled spring flushed shoots (leaf/shoot) during the 1st week of October. From the 4th and 5th leaves of the spring shoot, the representative samples were selected and gathered in each individual replicate in October of the two seasons. The samples were washed with tap and distilled water, then oven-dried at 80 °C until a constant weight was reached, and finely ground for the determination of total nitrogen (N), phosphorus (P), potassium (K), calcium (Ca), magnesium (Mg), iron (Fe), and zinc (Zn). The total N content in leaf was determined by the modified micro-Kjeldahl method mentioned in [56]; total leaf P was determined after wet digestion of plant ground leaves using sulfuric and perchloric acids, according to the method of [57]; total leaf K was determined photometrically in the digested material according to the method described by [58]. In addition, Ca and Mg percentages, as well as Fe, Mn, and zinc content, were determined using an atomic absorption spectrophotometer (Plasma-Optical Emission Spectrometry (Ultima2, Horiba Scientific, Unterhaching, Germany) according to [59].

#### 4.4.3. Productivity Measurements

At full flowering over the growing season, the number of optimum flowers per tagged limb was counted. Following the fall of 75%, the fruit set percentage of full flowers was calculated as follows:

(1) $$\mathrm{Fruit}\;\mathrm{set}\;\left(\%\right)=\frac{\mathrm{No}.\;\mathrm{of}\;\mathrm{set}\;\mathrm{fruitlets}}{\mathrm{No}.\;\mathrm{of}\;\mathrm{full}\;\mathrm{flowers}}\times 100$$

At a given date in December during each experimental season, the percentage of retained fruits was estimated according to the following equation:

(2) $$\mathrm{Fruit}\;\mathrm{retention}\;\left(\%\right)=\frac{\mathrm{No}.\;\mathrm{of}\;\mathrm{presented}\;\mathrm{fruits}\;\mathrm{at}\;a\;\mathrm{specific}\;\mathrm{date}}{\mathrm{No}.\;\mathrm{of}\;\mathrm{set}\;\mathrm{fruitlets}}\times 100$$

In late December 2019 and 2020, each tree’s fruits were collected separately, then numbered and weighed. The yield per hectare was computed by multiplying the production of the tree by the number of trees in a hectare.

#### 4.4.4. Fruit Quality

The physical characteristics of fruits such as weight, dimensions (equatorial and polar), shape index (length to width), volume, juice percentage, as well as diameter were measured in 15 fruits for all treatments were taken from the selected branches for each direction. Furthermore, fruit chemical properties as fruit juice and total soluble solids percentage (TSS %) were determined using a Carl Zeiss handheld refractometer. Fruit total acidity (g of citric acid per 100 mL of juice) and ascorbic acid (V.C.) content (mg of ascorbic acid per 100 mL of fruit juice) were determined according to [60]. The total soluble solids/acid ratio was also estimated. Ascorbic acid/vitamin C content was determined using a 2,6-dichlorophenol indophenol indicator for titration according to [60]. In addition, the total sugar percentage was determined according to the method described by [61]. At harvest time, samples of the treated fruits were collected and left at room conditions (20 °C ± 5 °C and 70–75% R.H.) and the number of days at which treated fruits retained a good appearance was counted, to determine shelf life.

### 4.5. Statistical Analysis

The data collected over two seasons in this work were subjected to analysis of variance according to [62]. Significant differences in means were also distinguished using Duncan’s multiple range test, with capital letters used to differentiate the means of different treatments for each investigated characteristic. Furthermore, principal component analysis (PCA), and clustered correlation heatmaps for yield and quality parameters were performed using scikit learn and seaborn functions in Python.

## 5. Conclusions

The results stated that the foliar spraying of Washington navel orange trees grown under similar environmental conditions and horticulture practices in the current experiment with Fe-NPs 2 and/or 0.1% Fe-chelated is a beneficial method for improving vegetative growth, nutritional status, fruiting aspects, and fruit quality. However, in most cases, the effects of the lower nano-Fe concentration used here were equivalent to the effects of the highest chelated iron concentration. Because of its lower absorption and efficiency, when compared with nano- and chelated forms, the mineral form of iron (FeSO_4_) demonstrated the lowest values of growth, yield, and quality. Nanoform of iron is recommended in arid regions to achieve higher yields with good quality and to be more environmentally friendly.

## Figures and Tables

**Table 8:** The evaluated treatments and their identifications.

No.	Treatment	Symbol
1	Control (spraying with tap water)	T1
2	Fe-NPs1 (1/80 dilution of the Fe-NPs stock solution)	T2
3	Fe-NPs2 (1/40 dilution of the Fe-NPs stock solution)	T3
4	0.1% ferrous sulphate (FeSO_4_·7H_2_O)	T4
5	0.2% ferrous sulphate (FeSO_4_·7H_2_O)	T5
6	0.05% Fe-chelated (EDTA)	T6
7	0.1% Fe-chelated (EDTA)	T7

## Data Availability

The data for this study are included in the main document and supplementary file.

## Supplementary Materials

The following are available online at https://www.mdpi.com/2223-7747/10/12/2577/s1, Supplementary Figure S1: SEM images of the prepared Fe-NP2, Supplementary Figure S2: TEM micrograph of the prepared Fe nanoparticles, Supplementary Figure S3: Zeta potential of Fe nanoparticle, Supplementary Figure S4: Dynamic light scattering, Supplementary Table S1: Soil physical and chemical properties before the first season.
