# Supplementary material for: Foliar Application of Nano, Chelated, and Conventional Iron Forms Enhanced Growth, Nutritional Status, Fruiting Aspects, and Fruit Quality of Washington Navel Orange Trees (Citrus sinensis L. Osbeck)
Source: Plants (Basel). 2021 Nov 25;10(12):2577. doi: 10.3390/plants10122577 (PMC8704722; doi:10.3390/plants10122577)
Supplement: Supplementary file 1 [file plants-10-02577-s001.zip › plants-1474386-supplementary.pdf]

Table S1. Soil physical and chemical properties prior to the first season 2019

Soil property	Value	Soil property		Value	
Particle size distribution (%)		Soluble ions ($mmol_c L^{-1}$)			
		Cations		Anions	
Coarse sand	11.0	Ca^{2+}	6.8	CO_3^{2-}	0.0
Fine sand	19.4	Mg^{2+}	3.2	HCO_3^-	4.5
Silt	20.2	Na^+	4.3	Cl^-	6.4
Clay	49.4	K^+	1. 8	SO_4^{2-}	5.2
Texture class*	Heavy Clay	Nutrients (mg kg^{-1})			
Soil pH (1:2:5 w/v – soil/water)	7.8				
EC (dS m^{-1})	1.65	N		119	
OM (%)	12	P		7.4	
Calcium carbonate (%)	23	K		57	
		Cu		2.3	
		Zn		4.3	
		Fe		2.2	

* texture class is according to international soil triangle; O.M: soil organic matter; EC: soil electrical conductivity

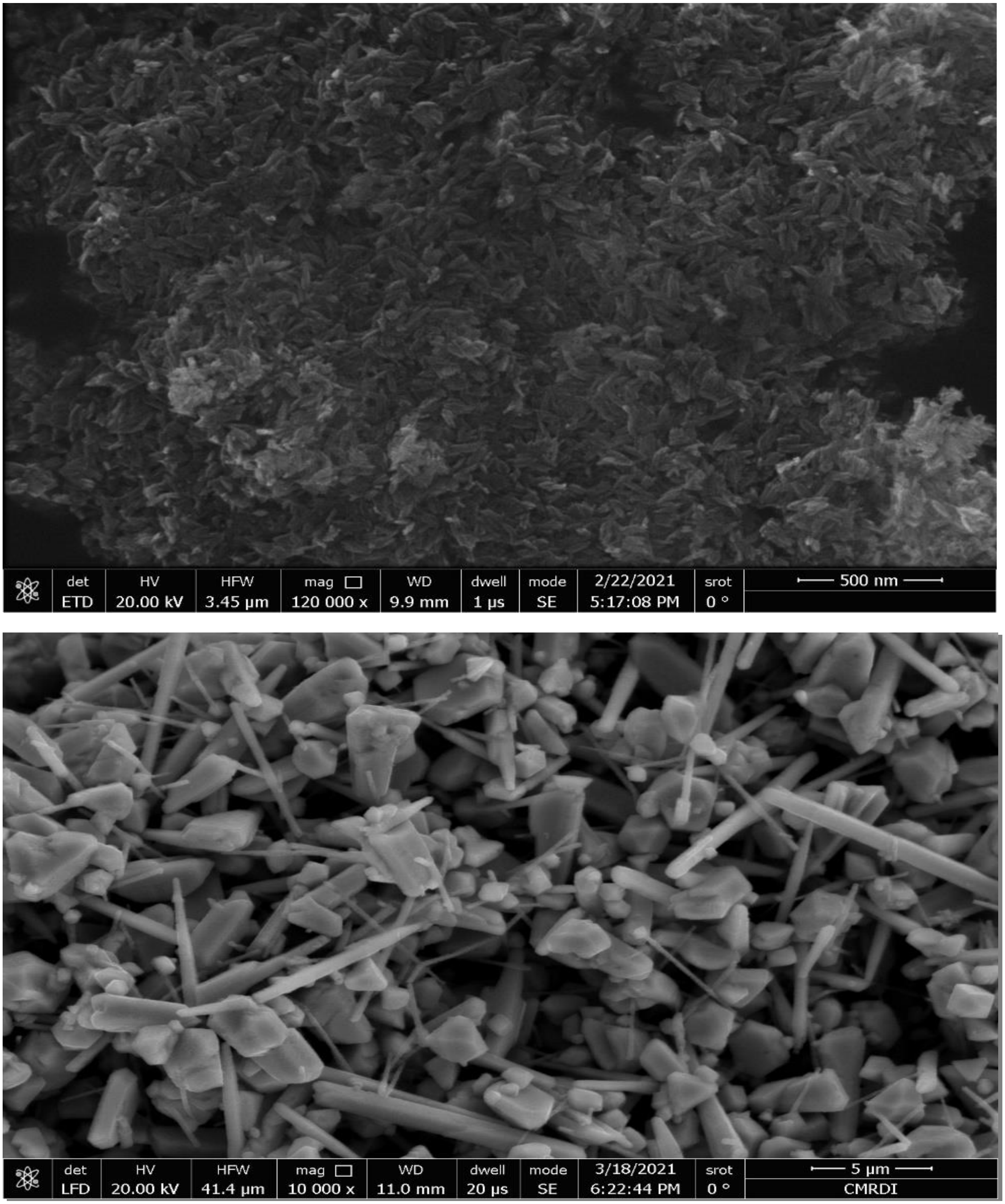

Figure S1. Scanning electron microscopy (SEM) images of the prepared Fe-NPs

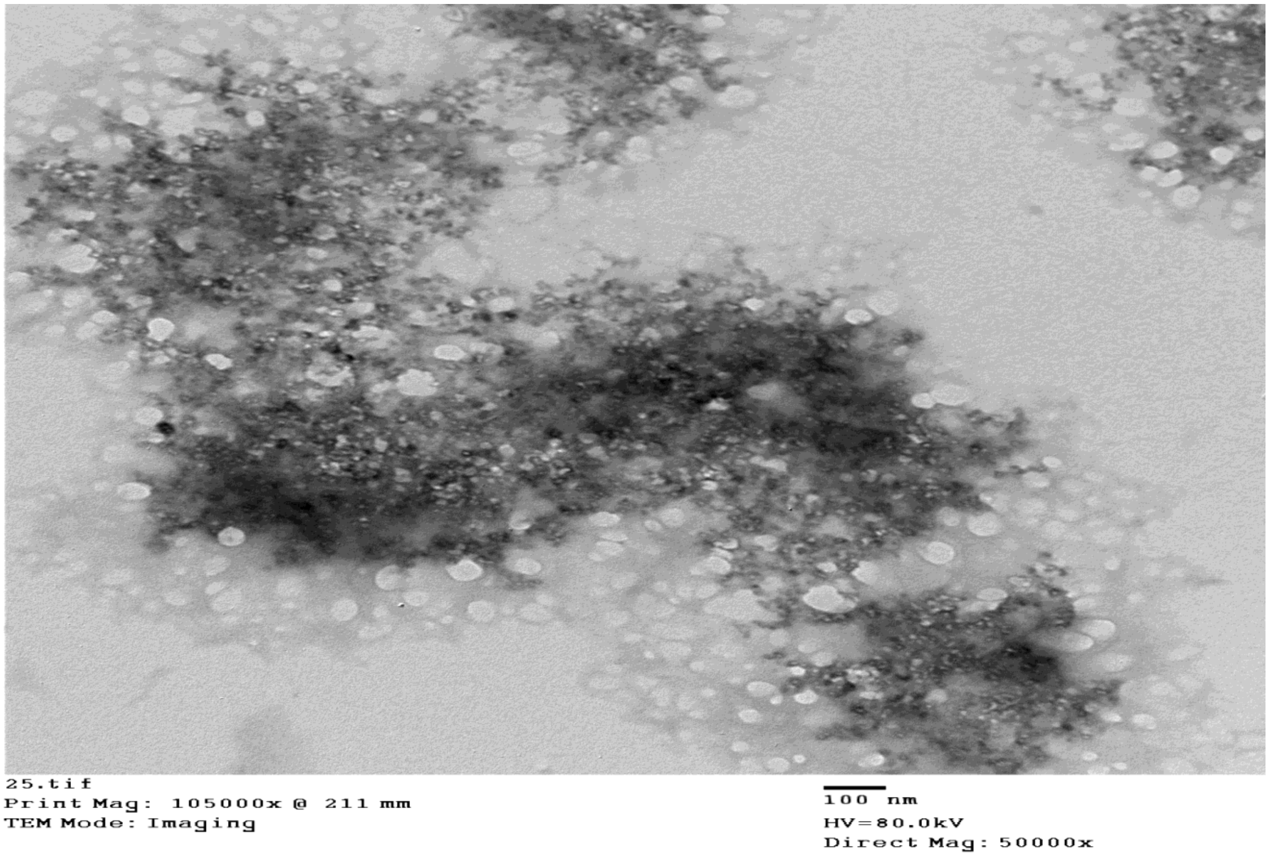
25.tif
Print Mag: 105000x @ 211 mm
TEM Mode: Imaging
100 nm
HV=80.0kV
Direct Mag: 50000x

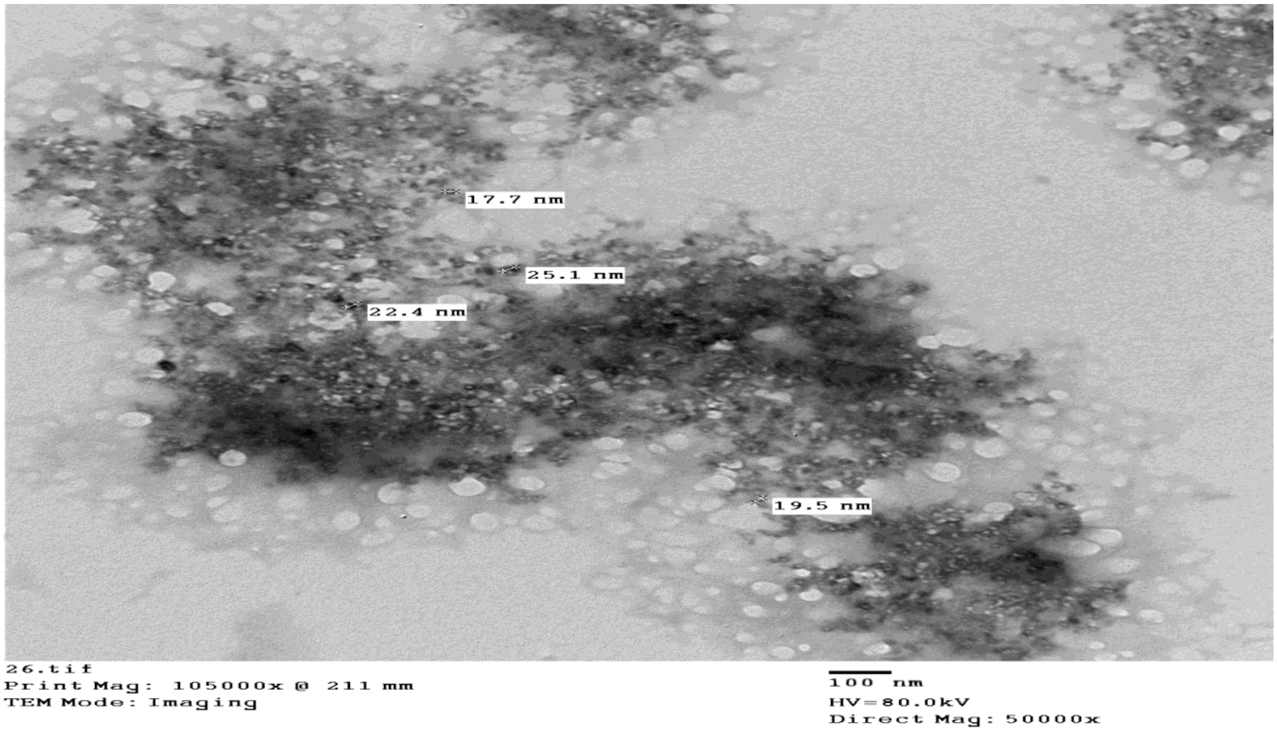

Figure S2 TEM micrograph of prepared Fe nanoparticles

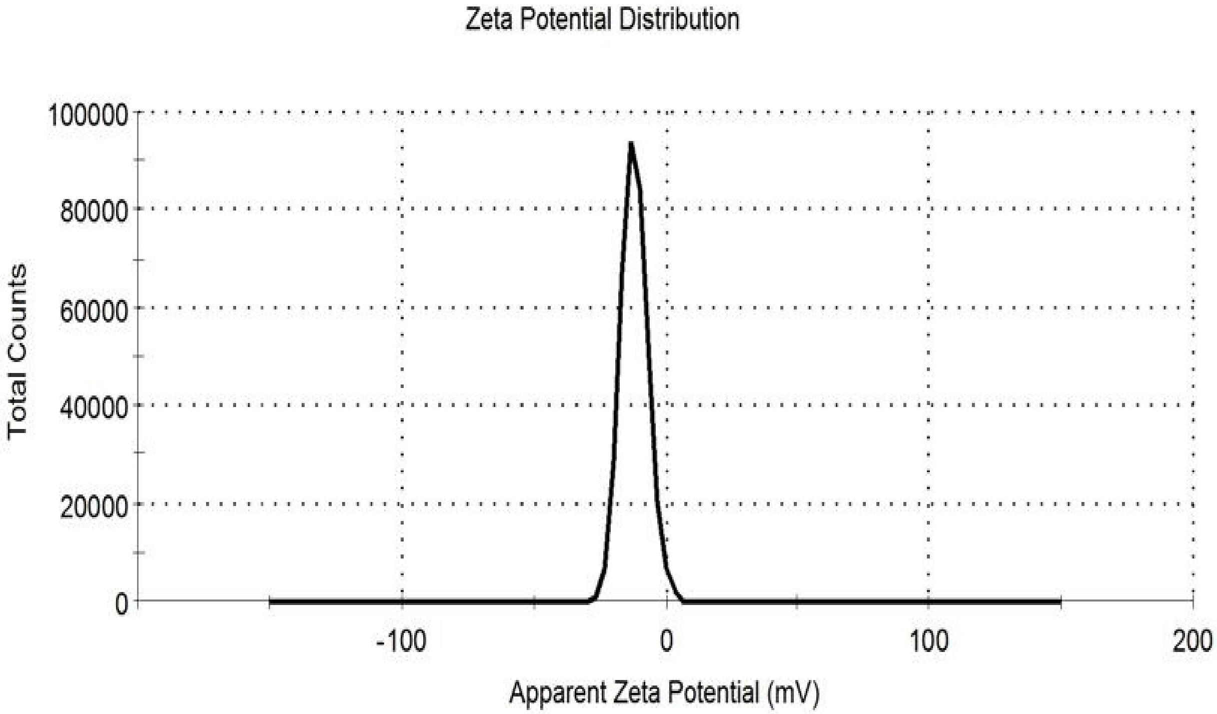

Figure S3. Zeta potential of the iron nanoparticles

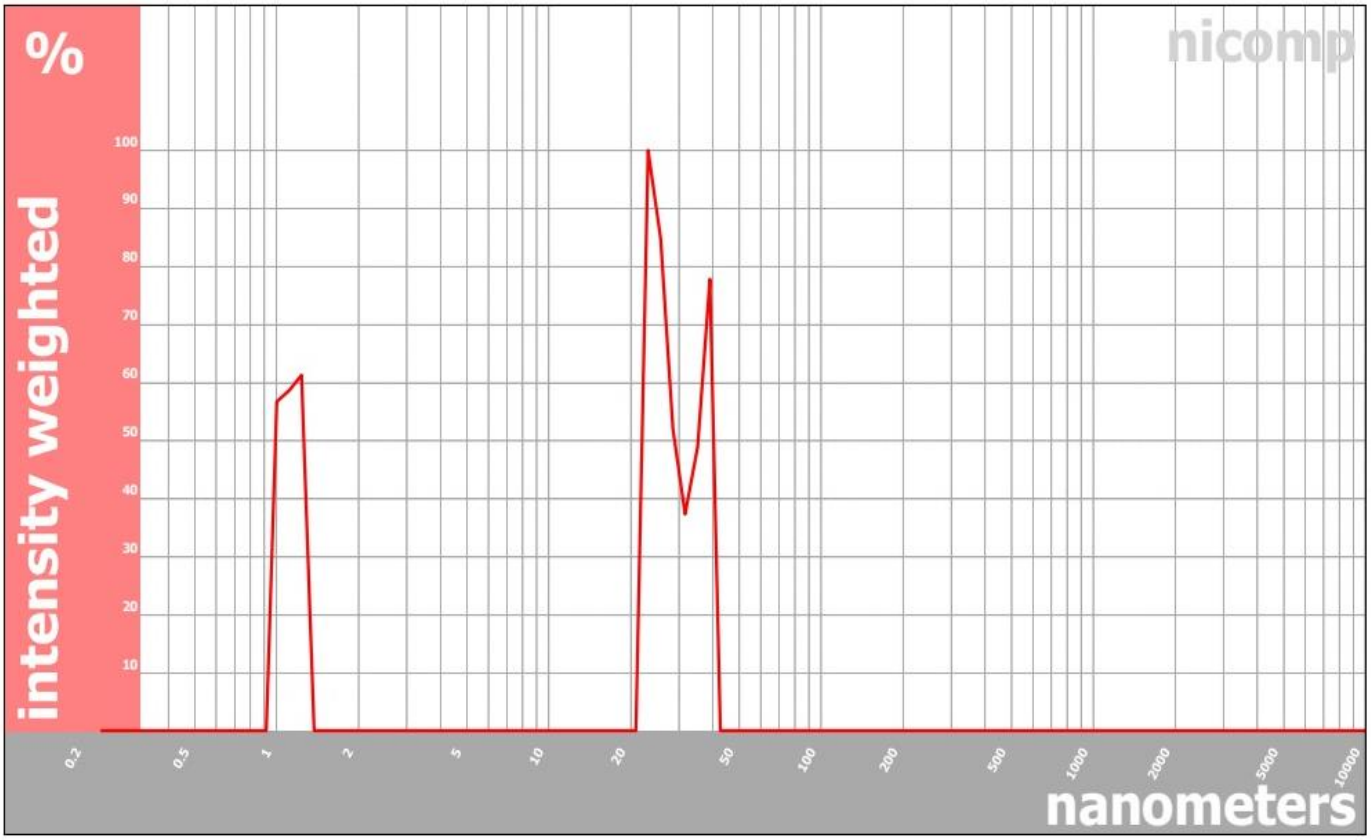

Figure S4. Dynamic light scattering (DLS)
